# Synergistic Adulticidal Activity of Lemongrass (*Cymbopogon citratus*), Star Anise (*Illicium verum*), Nutmeg (*Myristica fragrans*) Essential Oil and Their Components Against the Housefly (*Musca domestica*) and Their Safety for Key Non-Target Organisms

**DOI:** 10.3390/insects17040412

**Published:** 2026-04-10

**Authors:** Hataichanok Passara, Chamroon Laosinwattana, Tanapoom Moungthipmalai, Kouhei Murata, Mayura Soonwera

**Affiliations:** 1 Office of Administrative Interdisciplinary Program on Agricultural Technology, School of Agricultural Technology, King Mongkut’s Institute of Technology Ladkrabang, Ladkrabang, Bangkok 10520, Thailand; hataichanok.pa@kmitl.ac.th (H.P.); chamroon.la@kmitl.ac.th (C.L.); 64604012@kmitl.ac.th (T.M.); 2 School of Agriculture, Tokai University, Kumamoto 862-8652, Japan; kmurata@agri.u-tokai.ac.jp

**Keywords:** EO binary mixture, earthworms, guppy, *Musca domestica*, non-target honeybee, star anise + geranial, synergistic adulticides

## Abstract

Botanical insecticides containing a mixture of plant essential oils (EOs) are considered suitable for the management of houseflies (*M. domestica*). The adulticidal efficacies of single EOs and mixtures of EOs, including lemongrass (*C. citratus*), star anise (*I. verum*), nutmeg (*M. fragrans*), and their components (geranial, *trans*-anethole, and *α*-pinene), against houseflies were determined in comparison to 2% (*w*/*v*) *α*-cypermethrin as the positive control and distilled water as the negative control. The mixture of star anise EO (1%) + geranial (1%) was the most effective adulticide, superseding single EOs, other combinations of EOs, and its active component, *α*-cypermethrin, and distilled water. This mixture was highly synergistic and was found to be over 74% more toxic than all single EOs and almost 2.6 times more toxic than *α*-cypermethrin. Furthermore, the tested EOs did not cause mortality in guppies (*P. reticulata*) or earthworms (*E. fetida*), and caused a maximum of 48% mortality in honeybees (*A. mellifera*) at 24 h; by contrast, *α*-cypermethrin led to 100% mortality in honeybees within 0.5 h and in guppies and earthworms within 24 h, although it had low toxicity toward houseflies. Thus, a mixture of star anise EO + geranial is a promising source of EO-derived insecticides for housefly control that is also safe for important non-target species.

## Simple Summary

Houseflies (*Musca domestica*) are common insect pests that transmit diseases to humans and livestock. They have predominantly been controlled by means of chemical insecticides, which have become ineffective due to the development of resistance in houseflies. Moreover, insecticides have negative side effects on beneficial organisms and the environment. Therefore, safe and sustainable alternatives are immediately required. Plant essential oils are natural compounds that degrade rapidly and are generally considered less harmful than many synthetic chemicals. This study evaluated the kill potential of lemongrass (*Cymbopogon citratus*), star anise (*Illicium verum*), and nutmeg (*Myristica fragrans*) essential oils, as well as their principal components, against adult houseflies. These natural, plant-based treatments were compared to a widely used synthetic insecticide. A mixture of star anise oil and one of its major components emerged as the most effective treatment, killing significantly more flies than single oils and even surpassing the synthetic insecticide. Crucially, there were no detrimental effects of the plant-based treatments on honeybees (*Apis mellifera*), guppies (*Poecilia reticulata*), and earthworms (*Eisenia fetida*). By contrast, the chemical insecticide killed all tested beneficial organisms. These results demonstrate that certain plant oil mixtures are effective in managing houseflies while preventing harm to non-target species and the environment. They hold promise as safer alternatives for integrated pest management programs.

## 1. Introduction

Houseflies (*M. domestica*; Diptera: Muscidae) are one of the most common synanthropic pests worldwide [1,2]. They are highly effective mechanical vectors of a wide range of pathogens to humans, companion animals, and livestock facilities. As a consequence of its fast reproduction rate, short generation time, and high ecological plasticity, *M. domestica* has successfully colonized a wide range of environments and efficiently spread pathogens, causing myiasis, diarrheal diseases, dysentery, anthrax in livestock [3], lumpy skin disease [4] and Newcastle disease [5], inflicting severe public health and economic losses [6]. Over the years, housefly control has largely been dominated by chemical pesticides, including pyrethroids, organophosphates, and neonicotinoids. However, excessive and careless use of these insecticides has led to widespread housefly resistance, significantly compromising the efficacy of control and creating a demand for higher doses [7,8,9,10]. In addition to causing insect resistance, the synthetic compounds in these insecticides have commonly displayed environmental persistence, leading to concomitant contamination of soil and water bodies, potential bioaccumulation through food chains, and acute and/or chronic toxicity in non-target organisms. As such, many ecological and regulatory concerns have been raised [9,11,12].

Plant-based essential oils (EOs) and their primary bioactive components appear to be a suitable alternative in sustainable pest-control strategies for the following reasons. EOs are complex mixtures with a high proportion of terpenoids and phenylpropanoids, which are easily degraded in the environment; thus, they are fairly safe to mammals. The multi-targeted nature of their action in insects, including neurotoxicity (e.g., acetylcholinesterase inhibition and octopaminergic disruption), repellency, feeding deterrence, oviposition inhibition, and growth/development interference, makes the development of resistance less likely compared to contact synthetic insecticides, and those that act by inhalation or other routes of entry [13,14,15]. In the literature, it is reported that the synergistic action between an EO compound and one of its major components significantly increased insecticidal activity, thereby allowing for lower effective concentrations, reduced environmental burden, and higher cost/benefit ratios in IPM strategies [6,16,17,18,19]. However, knowledge of the comparative antimicrobial efficacy of essential oils and their major constituents in binary combinations against *M. domestica* remains relatively sparse, especially under standardized experimental protocols. Furthermore, knowledge of their concurrent ecological safety in several non-target taxa is limited [18,19].

Environmental selectivity is a key criterion for the development of a new generation of bio-insecticides. Pollinators such as the honeybee (*A. mellifera*; Hymenoptera: Apidae) are essential for global food security and ecosystem resilience; however, their populations are under threat from chemical exposure [20,21]. Freshwater organisms, such as guppies (*P. reticulata*; Cyprinodontiformes: Poeciliidae), are sensitive bioindicators of environmental integrity and play an essential role in the biological control of mosquitoes and houseflies. These non-target species have been used as models to understand the association between habitat quality and control of vector-borne diseases [4,22]. Soil invertebrates such as earthworms (*E. fetida*; Opisthopora: Lumbricidae) are ecosystem engineers; they improve soil structure, organic matter decomposition, and nutrient cycling, which are essential for sustainable agriculture [23,24,25,26]. These organisms were intentionally chosen to represent important ecological systems (pollinators, aquatic ecosystems, and correct ecosystems), allowing for a broad view of environmental selectivity [24,25]. Demonstrating negligible toxicity against these models is therefore key to validating the environmental friendliness of EOs as an alternative intervention.

The plant species were selected based on their previously described bioactive properties, abundance, and representation of different major chemical classes (aldehydes, phenylpropanoids, and monoterpenes), enabling a direct comparison of their insecticidal potential as well as interactions between the plant-derived compounds. Therefore, the present study was designed to focus on the adulticidal activity of single EOs and mixtures of EOs as well as their principal components from lemongrass (*C. citratus*; Poales: Poaceae), star anise (*I. verum*; Austrobaileyales: Schisandraceae), and nutmeg (*M. fragrans*; Magnoliales: Myristicacae); major components such as geranial, *trans*-anethole, and *α*-pinene were also assessed against *M. domestica*, with an emphasis on detecting synergistic, additive, or antagonistic interactions between compounds. Concurrently, we conducted a multi-species biosafety evaluation on *A. mellifera*, *P. reticulata*, and *E. fetida* to study ecological selectivity. By combining synergistic interaction analysis and cross-taxa safety validation, this work provides a more robust and environmentally friendly scientific basis for the development of EO-based insecticides as a novel agent for housefly management. We believe this to be one of the few studies to integrate both an interaction-based assessment of efficacy and a cross-species evaluation of biosafety under a single experimental framework to assess the essential oil-based control of *M. domestica*.

## 2. Materials and Methods

### 2.1. Essential Oils, Chemicals, and Tested Species

The chemicals, essential oils, and test organisms used in this study are listed in Table 1. The chemical compositions of essential oils from lemongrass (*C. citratus*), star anise (*I. verum*), and nutmeg (*M. fragrans*) are listed in Table 2. They were steam-distilled and processed according to the Hazard Analysis and Critical Control Point (HACCP) rules, and their major and minor components were determined using appropriate analytical methods.

### 2.2. Treatments

Based on our previous publications [4,6], the concentrations assigned to all single EOs and EO mixtures were anticipated to have a strong adulticidal effect while being relatively non-toxic to non-target species. The single EOs and mixtures of three EOs and their principal component were diluted with 70% (*v*/*v*) ethanol. The six equivalent monotherapies included a 1% concentration for EOs of nutmeg, lemongrass, and star anise, as well as pure α-pinene, geranial and trans-anethole. Six composite treatments were developed, including 1% nutmeg EO + 1% geranial; 1% nutmeg EO + 1% trans-anethole; 1% lemongrass EO + 1% α-pinene; 1% lemongrass EO + 1% trans-anethole; 1% star anise EO + 1% geranial; and 1% star anise EO + 1% α-pinene.

All samples were generated and filtered a day before use. The aliquots were stored in brown screw-top bottles (100 mL) and preserved at the following laboratory conditions: 25 °C, 71% RH, and an alternating light/dark period every 12 h.

### 2.3. Housefly (M. domestica) Rearing

Pupae of the housefly were obtained from the Entomology Laboratory, School of Agricultural Technology at KMITL in Bangkok, Thailand. They were reared in an insectary (310 × 310 × 310 mm) with 100 pupae per cage, and maintained at a temperature of 25 °C and humidity of 71% RH under a L:D photoperiod of 12:12 h. The pupae were not nourished with any food. They emerged as adults after three to four days of pupation. Adults were fed with a mixture of glucose + milk + distilled water at 1:1:8, as reported by Soonwera and Sittichok [30]. Adult assays were performed using 3- to 4-day-old adults [4,30].

### 2.4. Adulticidal Activity Assay

**Figure 1:**
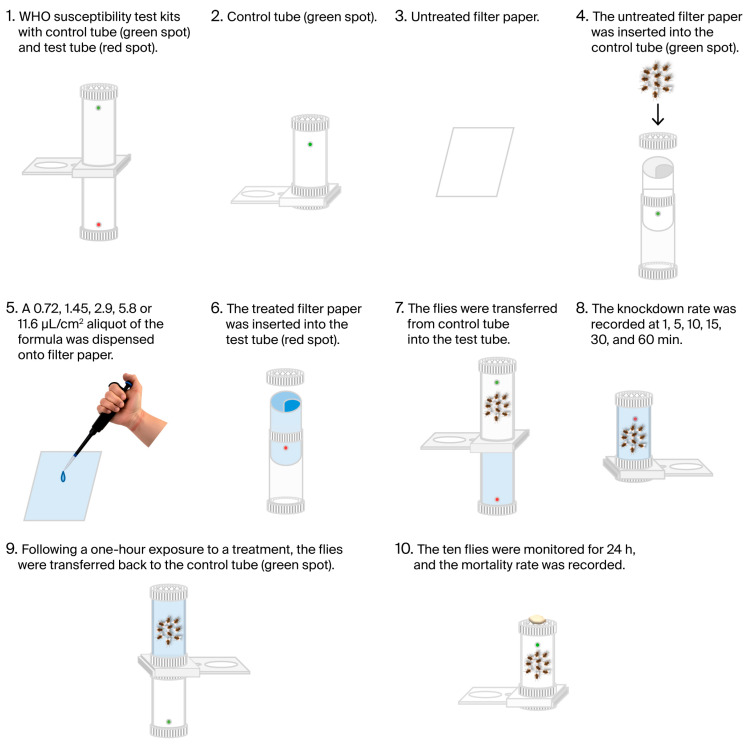
WHO standard susceptibility test for adulticidal activity assay.

The adulticidal efficacy against adult houseflies was tested using a modified WHO standard susceptible tube test. This method has been widely used for mosquito susceptibility assays and adapted in previous studies for other insect species (slightly modified for the housefly test) [30,31] (Figure 1). This approach allowed controlled exposure to treatments while monitoring mortality over time. Two test tubes (125 mm × 44 mm diameter) were employed, one with the treatment and the second initially containing a piece of intact filter paper. A total of 10 three-day-old flies (sex ratio 1:1) were exposed to 0.72, 1.45, 2.9, 5.8, or 11.6 μL/cm^2^ of a treatment test in the treatment tube (EOs were pipetted onto a 120 mm × 150 mm piece of filter paper) and held there for 1 h; they were then moved to the second tube and observed for an additional 24 h. A positive control (2% α-cypermethrin) and a negative control (distilled water) were used simultaneously. After 1 h of exposure, the subjects were provided with 10% syrup soaked onto cotton wool. Since no flies treated with the negative control died, the mortality of the treatments was not adjusted for the mortality in the negative control. The mortality rates of houseflies were recorded at 0.5, 1, 6, 12, and 24 h after exposure. Five replicates of each treatment were conducted, with 10 individuals per replicate (total = 50). The mortality of housefly adults for every treatment and control was calculated as follows [4,30]:

(1) $$\mathrm{Mortality}\;(\%M)=\left[\frac{\mathrm{MT}}{\mathrm{MN}}\right]\times 100$$

where MT is the number of dead flies, and MN is the number of treated flies.

After 24 h of exposure, the mortality index (MI) was determined as follows [21,30]:

(2) $$\mathrm{MI}=\frac{{\mathrm{LC}}_{50}\;\mathrm{of}\;\alpha \text{-}\mathrm{cypermethrin}}{{\mathrm{LC}}_{50}\;\mathrm{of}\;\mathrm{test}}$$

where the LC_50_ of *α*-cypermethrin is the 50% lethal concentration of *α*-cypermethrin, and the LC_50_ of the test is the 50% lethal concentration of the treatment. MI < 1 indicates that the formula was less harmful than *α*-cypermethrin; MI > 1 implies that the formula, either alone or in combination, was more toxic than *α*-cypermethrin.

Binary mixtures were more effective than single formulations, as indicated by their synergistic value (SV). SV was calculated as follows [21,30]:

(3) $$\mathrm{SV}=\frac{{\mathrm{LC}}_{50\;\mathrm{mix}}}{\left({\mathrm{LC}}_{50\;\mathrm{single}\;\mathrm{formula}\;1}+{\mathrm{LC}}_{50\;\mathrm{single}\;\mathrm{formula}\;2}\right)}$$

where the LC_50 mix_ is the median lethal concentration (50% lethal concentration) of the binary mixture formulation; LC_50 single formula 1_ is the median lethal concentration of formulation 1 assayed alone; and LC_50 single formula 2_ is the median lethal concentration of formulation 2 tested alone. An SV < 1 is indicative of synergistic interaction, and an SV ≥ 1 suggests an additive or antagonistic effect.

The following formula was used to derive the increased mortality value (IMV), which indicated that binary mixtures were more effective than single formulas [21,30].

(4) $$\mathrm{IMV}=[\frac{({\mathrm{LC}}_{50\;\mathrm{single}\;\mathrm{formula}\;1}+{\mathrm{LC}}_{50\;\mathrm{single}\;\mathrm{formula}\;2})-{\mathrm{LC}}_{50\;\mathrm{mix}}}{\left({\mathrm{LC}}_{50\;\mathrm{single}\;\mathrm{formula}\;1}+{\mathrm{LC}}_{50\;\mathrm{single}\;\mathrm{formula}\;2}\right)+{\mathrm{LC}}_{50\;\mathrm{mix}}}]\times 100$$

Here, the LC_50 mix_ is the median lethal concentration (50% lethal concentration) of the binary mixture formulation; LC_50 single formula 1_ is the median lethal concentration of formulation 1 assayed alone; and LC_50 single formula 2_ is the median lethal concentration of formulation 2 tested alone.

### 2.5. Safety Bioassay for Non-Target Species

For the honeybee bioassay, the lethal actions of single EOs and mixtures of EOs were tested against adult honeybees, following the method of Soonwera et al. 2024 [4,6]. In total, 700 honeybees were caged inside an insect cell (310 × 310 × 310 mm), with a population of 100 honeybees in each cage. The honeybees were kept in the laboratory for 3 days prior to the topical application assay (25.5 °C, 72.0% RH) under an alternating cycle of 12 h light and dark periods. They were fed a solution of fructose + distilled water in a proportion of 1:1, as described by Soonwera et al. 2024 [6]. The honeybees were cooled to −8 °C before topical administration. Then, three concentrations of each formulation (0.1, 0.5, and 1.0 mL/L) were prepared, and 1 µL of each test formulation was pipetted onto the mesonotum. After the application, 10 honeybees were placed into an 8.5 × 10 × 5.5 cm^3^ insect box and provided with a 50% (*w*/*v*) fructose solution. Each experiment was conducted in five replicates (each replicate included 10 honeybees), and negative (distilled water) and positive (*α*-cypermethrin) controls were included. Mortality was noted at 0.5, 1, 6, 12, and 24 h of exposure. The definition of mortality rate (%M) is as described in [4,6]:

(5) $$\mathrm{Mortality}\;(\%M)=\left[\frac{\mathrm{MB}}{\mathrm{MA}}\right]\times 100$$

where MB is the number of dead honeybees, and MA is the number of treated honeybees.

Guppies (*P. reticulata*) were purchased from a guppy farm at Bang Sao Thong District, Samut Prakan Province, Thailand (13°42′43.8″ N 100°47′41.7″ E). In line with Soonwera et al. (2024) [6] and Passara et al. (2024) [21], we evaluated the toxicity of treatments on guppies. A total of 2000 fish were bred in a 500 × 500 × 300 mm plastic container with clean water (85 L). Twelve hundred strong fish were selected and kept at 25.5 °C, 72% RH, in alternate periods of artificial light and dark (12 h of light and dark), pH 6.5–7.0, dissolved oxygen ≥ 5 mg/L, and water hardness of 75–100 mg/L. Both male and female guppies were used in the bioassay. Ten adult guppies were placed in a 5 L plastic container (diameter: 300 mm; height: 200 mm) filled with water. Three concentrations of every formulation, 0.1, 0.5, and 1.0 mL/L, were tested. Five replicates (each replicate included 10 fish) of each treatment concentration were evaluated simultaneously to the negative control (distilled water) and positive control (α-cypermethrin). Mortality was registered at 1, 3, 5, 7, and 14 days after treatment. Guppy mortality and unusual behavior associated with death were observed. Mortality rate (%M) was calculated as follows [6,21]:

(6) $$\mathrm{Mortality}\;(\%M)=\left[\frac{\mathrm{MB}}{\mathrm{MA}}\right]\times 100$$

where MB is the number of dead guppies, and MA is the number of treated guppies.

The procedure for toxicity testing against earthworms (*E. fetida*) followed the guidelines of the Organization for Economic Co-operation and Development (1984) [32] and Passara et al. (2024 and 2026) [21,26]. In total, 1000 earthworms were harvested on 16 December 2025 from a KMITL organic farm, Bangkok, Thailand and placed into a black plastic container (850 mm in diameter and 250 mm in height). Containers were filled with 5 kg of test soil (organic fertilizer, cow manure, coconut coir, and organic soil ratio = 1:1:1:1; pH level 6.5–7.0; and 65% of the soil moisture) [21,26], and the same conditions were used as the honeybee assay. We applied three concentrations (0.1, 0.5, and 1.0 mL/kg soil) of each treatment, thoroughly mixing the treatment with 1 kg of test soil for uniform distribution. The uniformity of essential oil distribution in the soil was evaluated by mixing the oil with a food-grade dye and visually assessing the color homogeneity. Consistent coloration throughout the soil sample was taken as evidence of uniform distribution. The treated soil was then transferred into black plastic containers (250 mm diameter × 200 mm height), and 10 earthworms were introduced into each container. Each treatment for the three concentrations was tested five times, along with the positive (2% *α*-cypermethrin) and negative control (distilled water). Mortality was monitored at 1, 3, 5, 7, and 14 d after treatment. Mortality rate (%M) (dead vs. total treated specimens) was computed in the same way as the honeybee and guppy bioassays.

The biosafety indices (BI) for honeybees, guppies, and earthworms were calculated as follows [4,21,26]:

(7) $$\mathrm{BI}=\frac{{\mathrm{LC}}_{50\;\mathrm{test}}}{{\mathrm{LC}}_{50\;\mathrm{cyper}}}$$

where LC_50 test_ is the 50% lethal concentration of treatments for non-target animals, and LC_50 cyper_ is the 50% lethal concentration of *α*-cypermethrin for non-target animals.

A BI of >1 implies that the EO treatment was relatively benign, whereas a BI of ≤1 implies that the treatment was relatively toxic to the non-target species.

### 2.6. Statistical Analysis

The statistical assumptions of the present study included uncorrelated observations, normal distribution, and homogeneity of variance. The bioassay was conducted according to a completely randomized design, with individuals randomly allocated to each replicate and treatment. All statistics were determined with IBM SPSS version 28 (Armonk, NY, USA). The mortality rates of all treatment bioassays were compared using a one-way ANOVA. The mean comparison testing across multiple treatments was performed with Tukey’s post hoc test (*p* < 0.05) [33]. The LC_25,50,90_ values of compounds were calculated using probit analysis [34] with IBM SPSS 27 and SAS 9.4. The sample size (the number of houseflies) used in each experiment was 10. All experiments were repeated five times. A sample size of 10 was assigned for honeybees, guppies, and earthworms. In all cases, individuals were randomly assigned to replicates and treatments for a fully comparable and unbiased experimental design.

## 3. Results

### 3.1. Adulticidal Activity Assay

**Figure 2:**
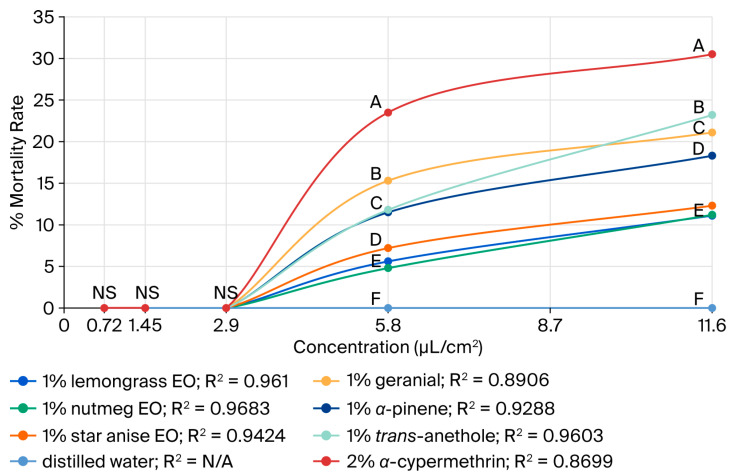
Mortality rate versus concentration for single EO formulas and *α*-cypermethrin against the adult houseflies (*M. domestica*) at 0.72, 1.45, 2.9, 5.8, and 11.6 µL/cm^2^. Values that are accompanied by different letters (A–F) denote significant differences between the treatments. (Tukey’s post hoc test, *p* < 0.05). NS indicates no significant difference.

**Figure 3:**
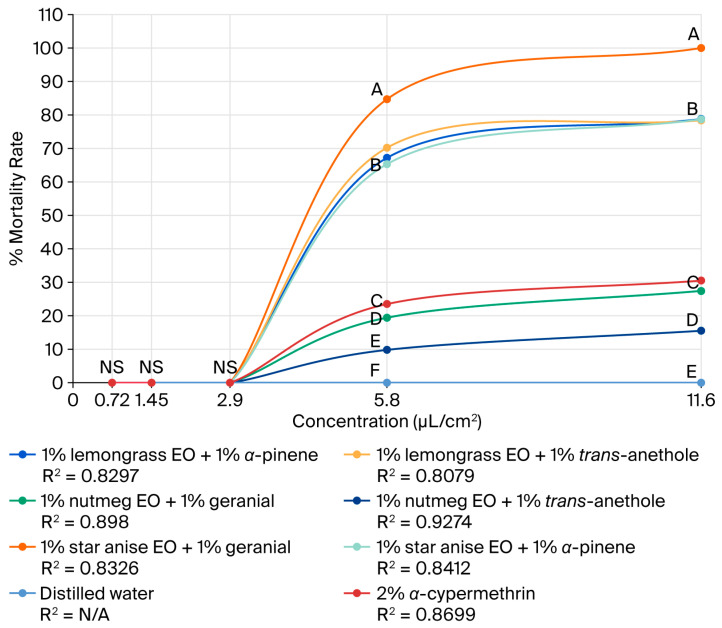
Mortality rate versus concentration for the binary mixture formulas and *α*-cypermethrin against the adult houseflies (*M. domestica*) at 0.72, 1.45, 2.9, 5.8, and 11.6 µL/cm^2^. Values that are accompanied by different letters (A–F) showed significant differences between treatments. (Tukey’s post hoc test, *p* < 0.05). NS indicates no significant difference.

**Figure 4:**
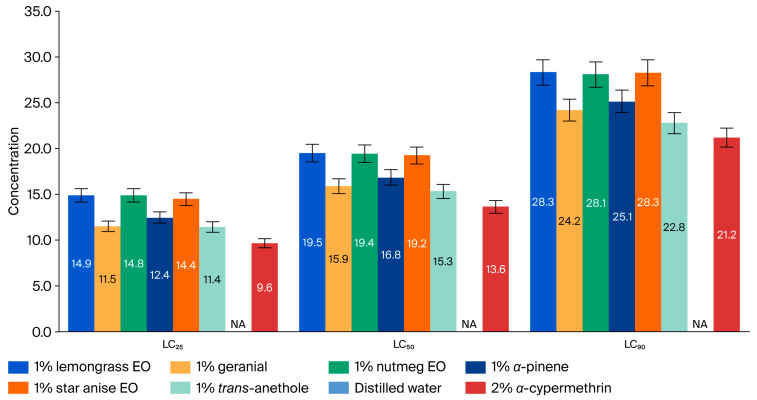
Concentrations of single formulations, distilled water, and *α*-cypermethrin that caused 25, 50, and 90% mortality in houseflies (*M. domestica*) after 24 h of exposure. LC_25,50,90_ = concentration required for 25%, 50%, and 90% mortality. NA indicates not available.

**Figure 5:**
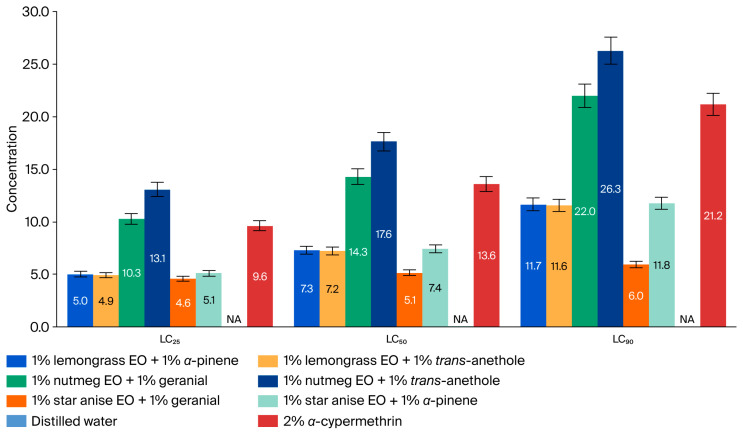
Concentration of binary mixtures, distilled water, and *α*-cypermethrin that caused 25, 50, and 90% mortality in houseflies (*M. domestica*) after 24 h of exposure. LC_25,50,90_ = concentration required for 25%, 50%, and 90% mortality. NA indicates not available.

**Figure 6:**
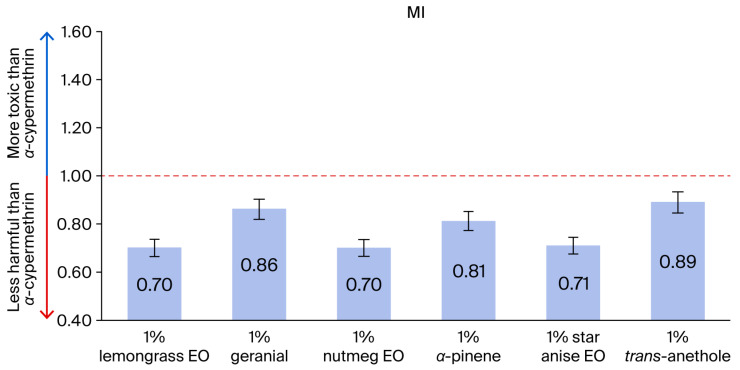
The mortality index (MI) of single-component formulations compared to *α*-cypermethrin. Note: MI < 1 indicates that the formula was less harmful than *α*-cypermethrin; MI > 1 suggests that the formula was more toxic than *α*-cypermethrin.

**Figure 7:**
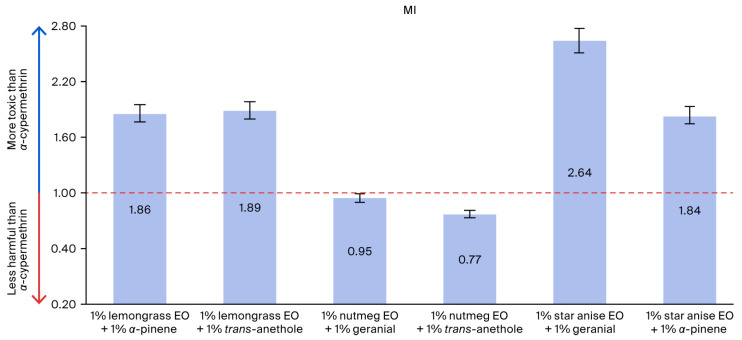
The mortality index (MI) of binary mixtures was compared to *α*-cypermethrin. Note: MI < 1 indicates that the formula was less harmful than *α*-cypermethrin; MI > 1 suggests that the formula was more toxic than *α*-cypermethrin.

**Figure 8:**
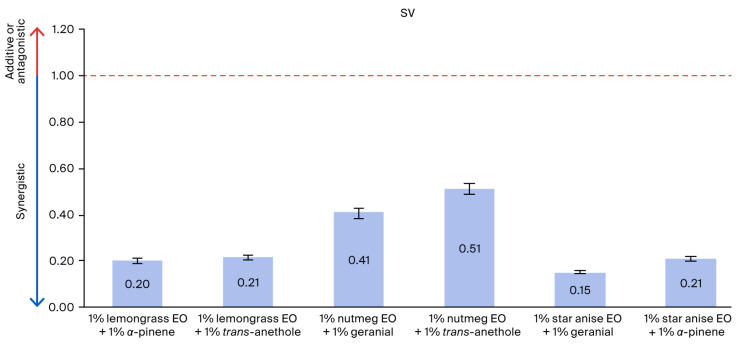
Synergistic value of binary mixtures against adult houseflies (*M. domestica*) versus corresponding single formulations.

**Figure 9:**
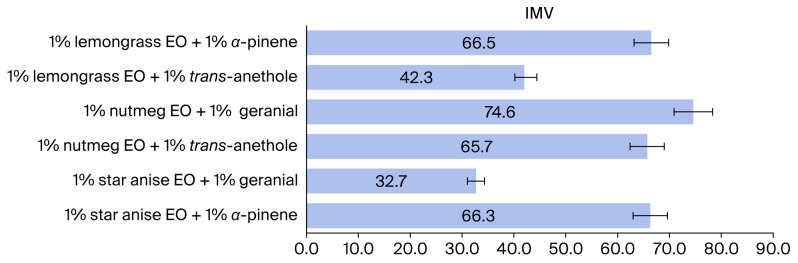
Increased mortality value (IMV, %) of binary mixtures against adult houseflies (*M. domestica*) versus corresponding single formulations.

The adulticidal activities of all tested formulations against houseflies are presented in Figure 2, Figure 3, Figure 4 and Figure 5, where the mortality rate is plotted against exposure time, and LC_50_ values are compared between single formulations, mixture formulations, and α-cypermethrin at dosage levels of 0.72, 1.45, 2.9, 5.8, or 11.6 µL/cm^2^ for each treatment, respectively. The most efficacious binary mixture formulation induced 100% mortality. On the other hand, death rates were 22% for single-component formulations and 30% for *α*-cypermethrin (2%). No deaths were observed for the negative control. These results demonstrate that the two-component mixture has significantly greater adulticidal activity compared to each active compound alone and the positive and negative controls. The LC_50_ values of the binary mixture formula varied between 5.1 and 17.6 µL/cm^2^. The mixture of 1% star anise essential oil and 1% geranial had the lowest LC_50_ value (5.1 µL/cm^2^). The LC_50_ values of the single-component formulations, on the other hand, ranged between 15.3 and 19.5 µL/cm^2^, which is relatively less toxic against adult houseflies. Two percent *α*-cypermethrin (positive control) had an LC_50_ of 13.6 µL/cm^2^ for comparison. It is worth mentioning that the best binary mixture was significantly more potent than *α*-cypermethrin. There was a remarkable synergistic effect between star anise essential oil and geranial against adult flies compared to single compounds. The mortality index (MI) values of the binary mixture formulation were compared to those of the single-component formulation against *α*-cypermethrin, as presented in Figure 6 and Figure 7. The MI values of the binary mixtures were between 0.77 and 2.64, and were higher than those of the single formulations (ranging from 0.70 to 0.89). An MI value of more than one suggests greater adulticidal toxicity than *α*-cypermethrin. Several binary mixture formulations (MI > 1) demonstrated greater adulticidal efficacy than *α*-cypermethrin, and the single-component formulations (MI < 1) exhibited lower toxicity relative to the reference insecticide, supporting the enhanced effectiveness of the binary mixtures over both single compounds and *α*-cypermethrin. The binary combinations exhibited significant synergistic effects (SV = 0.15–0.51), which were more effective than all essential oils (EOs) tested alone (Figure 8). An SV < 1 is indicative of synergistic interaction, and an SV ≥ 1 suggests an additive or antagonistic effect. As shown in Figure 9, the adulticidal efficacy of both mixture formulas was significantly superior when compared with each single EO formula evaluated alone. This resulted in an IMV between 32.7% and 74.6% more than single EO treatments.

### 3.2. Safety Bioassay for Non-Target Pollinators and Earthworms

The toxic effects of single EOs and their binary mixtures were investigated on three non-target organisms with habitats in various environmental locations (the honeybee, *A. mellifera* (air); the guppy, *P. reticulata* (water); and the earthworm, *E. fetida* (soil)). Toxicity at LC_50_ (µL/bee), adult mortality, and the Biosafety Index (BI) were evaluated after 24 h of exposure (Table 3, Table 4 and Table 5). Single and binary EO mixture formulations showed no lethal effect against non-target honeybees. The highest mortality rate was less than 48%, and the LC_50_ value ranged from 1.01 to 1.45 µL/bee, whereas BI varied between 8.42 and 12.08, demonstrating low toxicity and a good level of biosafety. In contrast, 2% *α*-cypermethrin resulted in the death of all adult pollinators (100%) 30 min after exposure, with a significantly lower LC_50_ value (0.12 µL/bee). This is indicative of high acute toxicity. Notably, the negative control did not kill any honeybees (Table 3). The single and binary EO combinations have a high safety profile for guppies and *P. reticulata*, and no mortality was observed during 24 h of exposure. No significant difference was observed compared to the control of distilled water, confirming the low acute toxicity of EOs towards fish. These results indicate that EO formulations would be of low risk to the freshwater environment and aquatic biodiversity under experimental study conditions. By contrast, the application of *α*-cypermethrin killed 100% of the fish within 24 h, with an extremely low LC_50_ (0.31 mL/L). These findings suggest that *α*-cypermethrin is a high environmental risk in the aquatic ecosystem (Table 4). The same tendency was also found in the earthworm *E. fetida*. No mortality was recorded in the EO mixture treatments, which showed similar activity to the distilled water control and a very safe profile for soil-related organisms. Considering the ecological relevance of earthworms in maintaining soil structure, cycling nutrients, and ensuring the overall fertility of soils, the lack of toxic effects indicates that the formulations based on EO are environmentally friendly. In contrast, *α*-cypermethrin caused 100% mortality at 24 h and had a low LC_50_ value of 0.75 mL/kg. This suggests that *α*-cypermethrin has high toxicity and causes risk to soil ecosystem health (Table 5).

## 4. Discussion

EOs are effective sources of natural pesticides, repellents, and growth inhibitors for controlling pests and other disease vectors of agricultural, medical, and veterinary importance [22,35]. They are effective for housefly pest control and offer great potential for the design of new adulticide alternatives because pesticidal activities have been reported in many cases [4,36,37]. They are relatively non-toxic to humans and several non-target pollinators, such as earthworms (*E. fetida*), but highly effective against houseflies (*M*. *domestica*) [4,30]. Some EO combinations were synergistic, which improved their effectiveness. However, most previous studies have evaluated such synergistic effects in isolation or on limited insect models, without integrating comparative assessments across multiple EO combinations and their major constituents using the same experimental framework, as conducted in the present study [33,38,39,40,41]. Accordingly, these EO mixtures show significant promise for development into a housefly adulticide. All four EO mixtures used in this study increased efficacy against adult flies. Their synergistic effect and mortality rate increased up to three times compared to those of single EO formulations. This magnitude of improvement is particularly relevant to practical applications, as it implies that an equivalent or a higher potency can be had at a lower concentration, yielding economic and environmental benefits. The most promising EO mixture in this study was 1% star anise (*I. verum*) EO + 1% geranial, which exhibited the greatest synergy and a mortality effect of up to 100% (LC50 = 5.1 µL/cm^2^). With respect to previously described mixtures of lemongrass (*C. citratus*) + star anise (*I. verum*) and geranial + *trans*-anethole, both were screened by the WHO tube susceptibility assay, which, despite having been originally developed for mosquitoes, has also been easily adapted for a variety of other insect species such as houseflies [10,42,43,44]. This adaptation enabled regulated exposure and precise tracking of mortality over time. The mortality of the adult flies was 100%, with an LT_50_ of <0.1 h after exposure for 24 h, which is in line with our present data [45]. Soonwera and Sittichok [30] revealed that the combination of 2.5% lemongrass EO (*C. citratus*) + 2.5% eucalyptus EO (*Eucalyptus globulus*) presented a mortality rate of 100% at 24 h and the highest synergistic value (99%). Two mixtures of citral + limonene and citral + geranyl acetate also exhibited potent activity against cabbage looper (*Trichoplusia ni*) larval and ovarian cell lines [46]. Sittichok et al. [43] found through a bioassay approach that a 1:1 blend of geranial + *trans*-cinnamaldehyde was highly synergistic against mosquito pupae (*Aedes aegypti*) as well as larvae, with an LT_50_ value in the range of 0.2 h. In this study, the combination of geranial and star anise showed strong synergism against houseflies. This finding is corroborated by several other studies [42,43,47], which demonstrated that star anise EO, *trans*-anethole, and geranial inhibit AChE activity in the nervous system of mosquitoes, corn weevils, and Mediterranean flour moths, leading to paralysis and death. In addition, the mixture of monoterpenes induced higher toxicity on the AChE of the mosquito nervous system than a single monoterpene [48]. Indeed, these observed synergistic interactions may be due to simultaneous action through multiple modes of action; increased penetration, metabolic disruption, or effects on different neural pathways may all additively contribute to overall toxicity in *M. domestica*.

Crucially, our LC_50_ values indicated that the EO mixture of 1% star anise (*I. verum*) EO + 1% geranial was over 2.6 times more potent than 2% *α*-cypermethrin. Likewise, the 1% dose of mixtures from other plants, such as 2.5% lemongrass (*C. citratus*) + 2.5% star anise (*I. verum*), 2.5% lemongrass (*C. citratus*) + 2.5% nutmeg (*M. fragrans*), and 2.5% nutmeg (*M. fragrans*) + 2.5% star anise (*I. verum*), has been reported to be 2.5 times stronger than the commercial insecticide (1%) *α*-cypermethrin against adult flies [49]. A combination of 1.0% geranial + 1.0% *trans*-anethole was also shown to be 246 times more potent against adult flies than the application of a commercial product containing 1% *w*/*v α*-cypermethrin [4]. These investigations established the fact that adult flies became resistant to α-cypermethrin. The additional enhanced performance of EO mixtures seen in this study further reinforces their potential as either resistance-breaking agents or effective options that can be utilized in integrated pest management programs. Our study results are in agreement with multiple other investigations, which demonstrated that *α*-cypermethrin exerts less efficacy against adults and has a high resistance rate. Zhang et al. [50] in China reported that in the periods of 2003–2005 to 2021–2022, insect pests became highly resistant to *β*-cypermethrin (with an increase from 14% of resistance to 26%). In another study, Abbas and Hafez [45] examined female houseflies of the 1st–23rd generations and identified that, though the cypermethrin concentration used was in the range of 90 to 1000 ppm during the entire study, the survival rate was higher at the end of study (74.8%) than the initial one (34.6%), which reflected a significant increase in resistance against cypermethrin. Resistance to adulticides involved detoxification enzymes. One review illustrates how the main families of detoxification enzymes, including cytochromes P450, carboxyl/cholinesterases, glutathione S-transferases (GST), and transporters, such as ATP-binding cassette (ABC) transporters, are implicated in insecticide resistance [51]. Taking into account multiple EO modes of action, resistance to EO is less likely to occur than to synthetic pesticides [52]. An 8.6-fold resistance to lavender EO vapor was detected in mature female bean weevils (*Acanthoscelides obtectus*) following eight generations of selective pressure [53]. Cross-resistance to synthetic insecticides may also explain the higher tolerance or resistance to EOs [54]. Such low mortality rates for individual EO treatments highlight the limited standalone adulticidal potential of single EOs. Their synergistic mixtures achieve a greater practical impact in field situations. While α-cypermethrin can inhibit acetylcholinesterase (AChE) and retard the breakdown of acetylcholine (ACh) in nerve tissue, it has become less effective against houseflies and even more toxic to non-targets such as honeybees and earthworms [55]. Most studies have established that cypermethrin is significantly toxic to pollinating bees and other beneficial arthropods [10,44]. It is especially toxic to honeybees, with a reported adult LD_50_ of 0.02–0.54 µg/bee, and a larval LC_50_ of around 1 mg/L [54,56]. Pashte and Patil [57] also identified an LC_50_ of 0.037% and an LT_50_ of 6.7 h for honeybees exposed to *α*-cypermethrin. The oral LD_50_ and contact LC_50_ for stingless bees (*Meliponula bocandei*) were 0.66–0.76 µg/mL and 92.24–223.69 µg/mL at 24–96 h, respectively [58]. Furthermore, *α*-cypermethrin has high mammalian acute oral neurotoxicity (oral LD_50_ 150–250 mg/kg) and may cause neurological, reproductive, developmental, and endocrine effects in vertebrates [59,60,61,62,63].

Conversely, we demonstrated that single EO formations and mixtures of EOs could be considered harmless to several bee species and earthworms at the experimental level. Significantly, this validation of multi-species safety enhances the ecological relevance of our findings as it simultaneously addresses the potential risks to multiple organisms, including pollinators and aquatic and soil invertebrates. Lemongrass, star anise, and geranial EOs resulted in <20% mortality rates and an LC_50_ of more than 1 µL/bee in honeybees and a BI of more than 10. A concentration of 1% *α*-cypermethrin produced mortality above 100%, with an LT_50_ of <0.08 h [4,6]. All together, these data suggest that the low mortality observed with single EO treatments limits their utility as standalone adulticidals; by contrast, binary EO mixtures show operationally meaningful and significantly higher efficacy, allowing more effective insect control with lower environmental impact and risk to non-target species. Crucially, there is a wealth of ecotoxicological evidence suggesting that, in contrast to broad-spectrum synthetic insecticides, many plant essential oils and their mixtures present a low acute risk to critical non-target organisms at concentrations that are effective against insect pests during pest management activities. Commercially available EO mixtures and compounds did not exert acute lethal toxicity to honeybees (*A. mellifera*) in laboratory trials. The biochemical impact was mostly sublethal even at a high dose [64]. Recent ecotoxicological examinations have revealed that formulations of essential oils from star anise (*I. verum*) and lemongrass (*C. citratus*), whose major bioactive constituents were *trans*-anethole and geranial, respectively, showed potent synergistic insecticidal activity but remained non-toxic to non-target aquatic organisms such as guppies (*P. reticulata*) and mollies (*P. latipinna*) in safety assays. These fish exhibited minimal mortality even after exposure to up to 10,000 ppm in the environment, while synthetic insecticides (DEET and *α*-cypermethrin) caused high toxicity under similar conditions [6,21]. Similarly, it is known that nutmeg (*M. fragrans*) contains phenylpropanoids such as myristicin and safrole, but it does not provoke lethal or sublethal effects against non-target aquatic fauna. As a corollary, no mortality was recorded in *P. reticulata* after exposure to the tested formulations for concentrations up to 1 mL/L. This adds to the evidence of the environmentally safe nature of plant-derived insecticides evaluated on non-target aquatic vertebrates. [65]. As for terrestrial soil fauna, the most recent studies have demonstrated that phenolic monoterpene-rich EOs have much higher LC_50_ values for earthworms (*E. fetida* and *E. eugeniae*) than standard pesticides, consistently highlighting that EOs are relatively less toxic to edaphic invertebrates [22,23,66,67]. Additionally, an EO mixture of star anise + geranial was not toxic to bees and earthworms. It is safe for human use without any negative poisoning side effects [68]. In addition, star anise EO has long been used in food and medicinal applications worldwide, especially in Asia (including Thailand) [68,69]. Geranial, a natural monoterpene aldehyde, is a constituent of many plant species, including lemongrass, citrus, and lemon. It has been widely used throughout Asian countries (Southeast Asia, China, and India) as well as South America for centuries [70,71]. These results are in line with those reported here. The formulations tested did not exhibit any visible adverse effect on the earthworms tested under experimental conditions, thus corroborating their lower toxicity against terrestrial soil invertebrates. Taken together, the data suggest that EO-based insecticidal mixtures have high potential for deployment with reduced ecological risk, as demonstrated by their selective toxicity towards target pests and limited effects on pollinators, freshwater vertebrates, and soil decayers, providing a good fit for scheduled pest control. To summarize, the plant-derived star anise-geranial combination exhibits a much higher additive or synergistic adulticidal activity against houseflies and a >6.8 times increase in the mortality of adult flies. In addition, mixed formulations were found to be non-toxic to several important non-target organisms (honeybees, guppies, and earthworms), making them good candidates for an EO-based alternative insecticide.

## 5. Conclusions

The combination of 1% star anise EO + 1% geranial showed a highly synergistic adulticidal effect against houseflies, with a low LC_50_ value (5.1 µL/cm^2^). In addition, no adverse effects were observed against several key non-target species, such as honeybees, guppies, and earthworms. This combination was about 6.8 times more effective than *α*-cypermethrin. Hence, this combination can be further developed as a spray-type EO insecticide for controlling houseflies both indoors and outdoors. In the future, the adulticidal efficacy of this EO mix in the field will be investigated. The stability of the product, its biosafety, potential sublethal effects, and behavioral changes in aquatic predators, humans, and mammals also require further investigation.

## Figures and Tables

**Table 1:** List of chemicals, essential oils, and tested species employed in this study.

Chemical	Source	Purity	Notes
*α*-pinene (CAS 80-56-8)	Sigma-Aldrich Company Limited, Saint Louis, MO, USA	98%	Technical grade
Geranial (CAS 5392-40-5)		96%	Technical grade
*trans*-anethole (CAS 4180-23-8)		99%	Technical grade
*α*-cypermethrin (positive control)	Agrichemical store in Bangkok, Thailand		10% (*w*/*w*)
Ethanol			95% (*v*/*v*)
Water (negative control)	Convenience store in Bangkok, Thailand		Distilled water
**Essential Oils**			
Lemongrass	Chemipan Corporation Co., Ltd., Rayong, Thailand	100%	
Star anise		100%	
Nutmeg		100%	
**Tested Organisms**			
Target			
Houseflies (*M. domestica*)	Collected from a local Ladkrabang market, Bangkok, Thailand	-	-
Non-Target Organisms			
Honeybee (*A. mellifera*)	School of Agricultural Technology, KMITL, Farm, Bangkok, Thailand	-	-
Guppy (*P. reticulata*)			
Earthworms (*E. fetida*)			

**Table 2:** The chemical composition of lemongrass (*C. citratus*) EO, star anise (*I. verum*) EO, and nutmeg (*M. fragrans*) EO.

No	Constituent	RI	KI	Relative Percentage of Total Composition			ID
				*C. citratus*	*I. verum*	*M. fragrans*	
1	Acetoin	680	680		0.2	-	RI, MS, Std
2	*α*-thujene	929	930	-	0.1	1.5	RI, MS, Std
3	*α*-pinene	935	936	3.5	0.1	24.3	RI, MS, Std
4	Camphene	946	946	-	-	0.4	RI, MS, Std
5	Sabinene	965	967	-	-	18.1	RI, MS, Std
6	*β*-pinene	971	970	-	-	15.1	RI, MS, Std
7	Myrcene	987	988	-	-	1.6	RI, MS, Std
8	*α*-phellandrene	1002	1006	-	-	0.5	RI, MS, Std
9	3-carene	1006	1005	-	0.2	1.5	RI, MS, Std
10	*α*-terpinene	1011	1010	0.2	0.1	2.5	RI, MS, Std
11	1,8-cineole	1025	1024	10.7	0.4	-	RI, MS, Std
12	Limonene	1028	1029	-	1.5	5.6	RI, MS, Std
13	O-cymene	1041	1041	-	0.2	1	RI, MS, Std
14	γ-terpinene	1051	1050	0.2	-	3.7	RI, MS, Std
15	Terpinolene	1080	1079	-	0.1	1.4	RI, MS, Std
16	Linalool	1085	1086	0.8	-	-	RI, MS, Std
17	*trans*-sabinene hydrate	1089	1087	-	-	0.2	RI, MS, Std
18	Terpinen-4-ol	1161	1162	-	-	4.1	RI, MS, Std
19	*α*-terpineol	1175	1175	-	0.4	0.5	RI, MS, Std
20	Neral	1216	1217	24.8	-	-	RI, MS
21	*p*-anisaldehyde	1222	1221	-	0.1	-	RI, MS, Std
22	Geraniol	1236	1237	4.7	-	-	RI, MS, Std
23	Geranial	1246	1247	46.1	-	-	RI, MS
24	Safrole	1269	1269	-	-	2.2	RI, MS, Std
25	*trans*-anethole	1284	1285	-	95.1	-	RI, MS, Std
26	Eugenol	1334	1335	-	0.5	0.3	RI, MS, Std
27	*α*-copaene	1374	1375	-	-	0.3	RI, MS
28	Geranyl acetate	1380	1380	4.1	-	-	RI, MS, Std
29	*cis*-isoeugenol	1410	1410	-	-	0.6	RI, MS, Std
30	Myristicin	1494	1493	-	-	13.2	RI, MS, Std
31	Elemicin	1522	1521	-	-	0.3	RI, MS
32	Caryophyllene oxide	1580	1581	2.1	-	-	RI, MS, Std
	Total identified (%)			97.2	99.0	98.9	

Compounds are listed according to their order of elution from the HP-5MS column. Linear retention index (RI) of HP-5 MS column, experimentally determined using standard alkanes (C7–C30). Kovat retention index (KI) from Babushok et al. (2011) [27] and National Library of Medicine (NLM) data library (https://pubchem.ncbi.nlm.nih.gov), accessed on 1 January 2026. Identification method (ID): RI values of the detected substances were matched to the corresponding values reported by Adams (2007) [28]; NIST 17 (2017) [29] and NLM library; MS: mass spectrum matching was performed with respect to Adams (2007) and NIST 17 (2017) [28,29]; Std: substance matching was performed with a readily available analytical standard (Sigma-Aldrich).

**Table 3:** Toxicity of treatment with EOs and *α*-cypermethrin against honeybees (*A. mellifera*) 24 h after exposure.

Treatment	Concentration (µL/Bee)	Mortality Rate (%) (Mean ± SD)					LC_50_ (µL/Bee)	BI
		0.5 h	1 h	6 h	12 h	24 h		
1% lemongrass EO	0.1	0 ^c^	0 ^c^	0 ^c^	0 ^c^	0 ^d^	1.33	11.08
	0.5	0 ^c^	0 ^c^	0 ^c^	0 ^c^	0 ^d^		
	1	0 ^c^	0 ^c^	0 ^c^	0 ^c^	8 ± 0.45 ^c^		
1% nutmeg EO	0.1	0 ^c^	0 ^c^	0 ^c^	0 ^c^	0 ^d^	1.38	11.50
	0.5	0 ^c^	0 ^c^	0 ^c^	0 ^c^	0 ^d^		
	1	0 ^c^	0 ^c^	0 ^c^	0 ^c^	6 ± 0.55 ^c^		
1% star anise EO	0.1	0 ^c^	0 ^c^	0 ^c^	0 ^c^	0 ^d^	1.33	11.08
	0.5	0 ^c^	0 ^c^	0 ^c^	0 ^c^	0 ^d^		
	1	0 ^c^	0 ^c^	0 ^c^	0 ^c^	8 ± 0.84 ^c^		
1% geranial	0.1	0 ^c^	0 ^c^	0 ^c^	0 ^c^	0 ^d^	1.26	10.50
	0.5	0 ^c^	0 ^c^	0 ^c^	0 ^c^	0 ^d^		
	1	0 ^c^	0 ^c^	0 ^c^	0 ^c^	12 ± 0.45 ^c^		
1% *α*-pinene	0.1	0 ^c^	0 ^c^	0 ^c^	0 ^c^	0 ^d^	1.29	10.75
	0.5	0 ^c^	0 ^c^	0 ^c^	0 ^c^	0 ^d^		
	1	0 ^c^	0 ^c^	0 ^c^	0 ^c^	10 ± 0 ^c^		
1% *trans*-anethole	0.1	0 ^c^	0 ^c^	0 ^c^	0 ^c^	0 ^d^	1.26	10.50
	0.5	0 ^c^	0 ^c^	0 ^c^	0 ^c^	0 ^d^		
	1	0 ^c^	0 ^c^	0 ^c^	0 ^c^	12 ± 1.1 ^c^		
1% lemongrass EO + 1% *α*-pinene	0.1	0 ^c^	0 ^c^	0 ^c^	0 ^c^	0 ^d^	1.01	8.42
	0.5	0 ^c^	0 ^c^	0 ^c^	0 ^c^	0 ^d^		
	1	0 ^c^	0 ^c^	0 ^c^	0 ^c^	48 ± 1.3 ^b^		
1% lemongrass EO + 1% *trans*-anethole	0.1	0 ^c^	0 ^c^	0 ^c^	0 ^c^	0 ^d^	1.21	10.08
	0.5	0 ^c^	0 ^c^	0 ^c^	0 ^c^	0 ^d^		
	1	0 ^c^	0 ^c^	0 ^c^	0 ^c^	16 ± 0.9 ^c^		
1% nutmeg EO + 1% geranial	0.1	0 ^c^	0 ^c^	0 ^c^	0 ^c^	0 ^d^	1.29	10.75
	0.5	0 ^c^	0 ^c^	0 ^c^	0 ^c^	0 ^d^		
	1	0 ^c^	0 ^c^	0 ^c^	0 ^c^	10 ± 0.7 ^c^		
1% nutmeg EO + 1% *trans*-anethole	0.1	0 ^c^	0 ^c^	0 ^c^	0 ^c^	0 ^d^	1.45	12.08
	0.5	0 ^c^	0 ^c^	0 ^c^	0 ^c^	0 ^d^		
	1	0 ^c^	0 ^c^	0 ^c^	0 ^c^	4 ± 0.55 ^c^		
1% star anise EO + 1% geranial	0.1	0 ^c^	0 ^c^	0 ^c^	0 ^c^	0 ^d^	1.29	10.75
	0.5	0 ^c^	0 ^c^	0 ^c^	0 ^c^	0 ^d^		
	1	0 ^c^	0 ^c^	0 ^c^	0 ^c^	10 ± 0 ^c^		
1% star anise EO + 1% *α*-pinene	0.1	0 ^c^	0 ^c^	0 ^c^	0 ^c^	0 ^d^	1.09	9.08
	0.5	0 ^c^	0 ^c^	0 ^c^	0 ^c^	0 ^d^		
	1	0 ^c^	0 ^c^	0 ^c^	0 ^c^	30 ± 1.22 ^b^		
Distilled water	0.1	0 ^c^	0 ^c^	0 ^c^	0 ^c^	0 ^d^	N/A	-
	0.5	0 ^c^	0 ^c^	0 ^c^	0 ^c^	0 ^d^		
	1	0 ^c^	0 ^c^	0 ^c^	0 ^c^	0 ^d^		
2% *α*-cypermethrin	0.1	0 ^c^	0 ^c^	50 ± 0.7 ^b^	88 ± 0.84 ^b^	90 ± 1 ^a^	0.12	-
	0.5	38 ± 0.84 ^b^	100 ^a^	100 ^a^	100 ^a^	100 ^a^		
	1	100 ^a^	100 ^a^	100 ^a^	100 ^a^	100 ^a^		
	ANOVA*_F_*_0.01_, D*f_total_*	**, 209	**, 209	**, 209	**, 209	**, 209		
	*p*-value	*p* < 0.01	*p* < 0.01	*p* < 0.01	*p* < 0.01	*p* < 0.01		

Mortality rates within a column where the same letters (a–d) do not differ significantly between the treatments (Tukey’s post hoc test, *p* < 0.01). ** for *p* < 0.01; N/A = not available.

**Table 4:** Toxicity of EO treatments and *α*-cypermethrin against guppies (*P. reticulata*) at 1, 3, 5, 7, and 14 days after exposure.

Treatment	Concentration (mL/L)	Mortality Rate (%) (Mean ± SD)					LC_50_ (mL/L)	BI
		1 Day	3 Days	5 Days	7 Days	14 Days		
1% lemongrass EO	0.1	0 ^b^	0 ^b^	0 ^b^	0 ^b^	0 ^b^	N/A	-
	0.5	0 ^b^	0 ^b^	0 ^b^	0 ^b^	0 ^b^		
	1	0 ^b^	0 ^b^	0 ^b^	0 ^b^	0 ^b^		
1% nutmeg EO	0.1	0 ^b^	0 ^b^	0 ^b^	0 ^b^	0 ^b^	N/A	-
	0.5	0 ^b^	0 ^b^	0 ^b^	0 ^b^	0 ^b^		
	1	0 ^b^	0 ^b^	0 ^b^	0 ^b^	0 ^b^		
1% star anise EO	0.1	0 ^b^	0 ^b^	0 ^b^	0 ^b^	0 ^b^	N/A	-
	0.5	0 ^b^	0 ^b^	0 ^b^	0 ^b^	0 ^b^		
	1	0 ^b^	0 ^b^	0 ^b^	0 ^b^	0 ^b^		
1% geranial	0.1	0 ^b^	0 ^b^	0 ^b^	0 ^b^	0 ^b^	N/A	-
	0.5	0 ^b^	0 ^b^	0 ^b^	0 ^b^	0 ^b^		
	1	0 ^b^	0 ^b^	0 ^b^	0 ^b^	0 ^b^		
1% *α*-pinene	0.1	0 ^b^	0 ^b^	0 ^b^	0 ^b^	0 ^b^	N/A	-
	0.5	0 ^b^	0 ^b^	0 ^b^	0 ^b^	0 ^b^		
	1	0 ^b^	0 ^b^	0 ^b^	0 ^b^	0 ^b^		
1% *trans*-anethole	0.1	0 ^b^	0 ^b^	0 ^b^	0 ^b^	0 ^b^	N/A	-
	0.5	0 ^b^	0 ^b^	0 ^b^	0 ^b^	0 ^b^		
	1	0 ^b^	0 ^b^	0 ^b^	0 ^b^	0 ^b^		
1% lemongrass EO + 1% *α*-pinene	0.1	0 ^b^	0 ^b^	0 ^b^	0 ^b^	0 ^b^	N/A	-
	0.5	0 ^b^	0 ^b^	0 ^b^	0 ^b^	0 ^b^		
	1	0 ^b^	0 ^b^	0 ^b^	0 ^b^	0 ^b^		
1% lemongrass EO + 1% *trans*-anethole	0.1	0 ^b^	0 ^b^	0 ^b^	0 ^b^	0 ^b^	N/A	-
	0.5	0 ^b^	0 ^b^	0 ^b^	0 ^b^	0 ^b^		
	1	0 ^b^	0 ^b^	0 ^b^	0 ^b^	0 ^b^		
1% nutmeg EO + 1% geranial	0.1	0 ^b^	0 ^b^	0 ^b^	0 ^b^	0 ^b^	N/A	-
	0.5	0 ^b^	0 ^b^	0 ^b^	0 ^b^	0 ^b^		
	1	0 ^b^	0 ^b^	0 ^b^	0 ^b^	0 ^b^		
1% nutmeg EO + 1% *trans*-anethole	0.1	0 ^b^	0 ^b^	0 ^b^	0 ^b^	0 ^b^	N/A	-
	0.5	0 ^b^	0 ^b^	0 ^b^	0 ^b^	0 ^b^		
	1	0 ^b^	0 ^b^	0 ^b^	0 ^b^	0 ^b^		
1% star anise EO + 1% geranial	0.1	0 ^b^	0 ^b^	0 ^b^	0 ^b^	0 ^b^	N/A	-
	0.5	0 ^b^	0 ^b^	0 ^b^	0 ^b^	0 ^b^		
	1	0 ^b^	0 ^b^	0 ^b^	0 ^b^	0 ^b^		
1% star anise EO + 1% *α*-pinene	0.1	0 ^b^	0 ^b^	0 ^b^	0 ^b^	0 ^b^	N/A	-
	0.5	0 ^b^	0 ^b^	0 ^b^	0 ^b^	0 ^b^		
	1	0 ^b^	0 ^b^	0 ^b^	0 ^b^	0 ^b^		
Distilled water	0.1	0 ^b^	0 ^b^	0 ^b^	0 ^b^	0 ^b^	N/A	-
	0.5	0 ^b^	0 ^b^	0 ^b^	0 ^b^	0 ^b^		
	1	0 ^b^	0 ^b^	0 ^b^	0 ^b^	0 ^b^		
2% *α*-cypermethrin	0.1	0 ^b^	0 ^b^	0 ^b^	0 ^b^	0 ^b^	0.31	-
	0.5	0 ^b^	100 ^a^	100 ^a^	100 ^a^	100 ^a^		
	1	100 ^a^	100 ^a^	100 ^a^	100 ^a^	100 ^a^		
	ANOVA*_F_*_0.01_, D*f_total_*	**, 209	**, 209	**, 209	**, 209	**, 209		
	*p*-value	*p* < 0.01	*p* < 0.01	*p* < 0.01	*p* < 0.01	*p* < 0.01		

Mortality rates within a column with the same letters (a, b) do not differ significantly between the treatments (Tukey’s post hoc test, *p* < 0.01). ** for *p* < 0.01; N/A = not available.

**Table 5:** Toxicity of treatments with EOs and *α*-cypermethrin against earthworms (*E. fetida*) at 1, 3, 5, 7, and 14 days after exposure.

Treatment	Concentration (mL/kg)	Mortality Rate (%) (Mean ± SD)					LC_50_ (mL/kg)	BI
		1 Day	3 Days	5 Days	7 Days	14 Days		
1% lemongrass EO	0.1	0 ^b^	0 ^b^	0 ^b^	0 ^b^	0 ^b^	N/A	-
	0.5	0 ^b^	0 ^b^	0 ^b^	0 ^b^	0 ^b^		
	1	0 ^b^	0 ^b^	0 ^b^	0 ^b^	0 ^b^		
1% nutmeg EO	0.1	0 ^b^	0 ^b^	0 ^b^	0 ^b^	0 ^b^	N/A	-
	0.5	0 ^b^	0 ^b^	0 ^b^	0 ^b^	0 ^b^		
	1	0 ^b^	0 ^b^	0 ^b^	0 ^b^	0 ^b^		
1% star anise EO	0.1	0 ^b^	0 ^b^	0 ^b^	0 ^b^	0 ^b^	N/A	-
	0.5	0 ^b^	0 ^b^	0 ^b^	0 ^b^	0 ^b^		
	1	0 ^b^	0 ^b^	0 ^b^	0 ^b^	0 ^b^		
1% geranial	0.1	0 ^b^	0 ^b^	0 ^b^	0 ^b^	0 ^b^	N/A	-
	0.5	0 ^b^	0 ^b^	0 ^b^	0 ^b^	0 ^b^		
	1	0 ^b^	0 ^b^	0 ^b^	0 ^b^	0 ^b^		
1% *α*-pinene	0.1	0 ^b^	0 ^b^	0 ^b^	0 ^b^	0 ^b^	N/A	-
	0.5	0 ^b^	0 ^b^	0 ^b^	0 ^b^	0 ^b^		
	1	0 ^b^	0 ^b^	0 ^b^	0 ^b^	0 ^b^		
1% *trans*-anethole	0.1	0 ^b^	0 ^b^	0 ^b^	0 ^b^	0 ^b^	N/A	-
	0.5	0 ^b^	0 ^b^	0 ^b^	0 ^b^	0 ^b^		
	1	0 ^b^	0 ^b^	0 ^b^	0 ^b^	0 ^b^		
1% lemongrass EO + 1% *α*-pinene	0.1	0 ^b^	0 ^b^	0 ^b^	0 ^b^	0 ^b^	N/A	-
	0.5	0 ^b^	0 ^b^	0 ^b^	0 ^b^	0 ^b^		
	1	0 ^b^	0 ^b^	0 ^b^	0 ^b^	0 ^b^		
1% lemongrass EO + 1% *trans*-anethole	0.1	0 ^b^	0 ^b^	0 ^b^	0 ^b^	0 ^b^	N/A	-
	0.5	0 ^b^	0 ^b^	0 ^b^	0 ^b^	0 ^b^		
	1	0 ^b^	0 ^b^	0 ^b^	0 ^b^	0 ^b^		
1% nutmeg EO + 1% geranial	0.1	0 ^b^	0 ^b^	0 ^b^	0 ^b^	0 ^b^	N/A	-
	0.5	0 ^b^	0 ^b^	0 ^b^	0 ^b^	0 ^b^		
	1	0 ^b^	0 ^b^	0 ^b^	0 ^b^	0 ^b^		
1% nutmeg EO + 1% *trans*-anethole	0.1	0 ^b^	0 ^b^	0 ^b^	0 ^b^	0 ^b^	N/A	-
	0.5	0 ^b^	0 ^b^	0 ^b^	0 ^b^	0 ^b^		
	1	0 ^b^	0 ^b^	0 ^b^	0 ^b^	0 ^b^		
1% star anise EO + 1% geranial	0.1	0 ^b^	0 ^b^	0 ^b^	0 ^b^	0 ^b^	N/A	-
	0.5	0 ^b^	0 ^b^	0 ^b^	0 ^b^	0 ^b^		
	1	0 ^b^	0 ^b^	0 ^b^	0 ^b^	0 ^b^		
1% star anise EO + 1% *α*-pinene	0.1	0 ^b^	0 ^b^	0 ^b^	0 ^b^	0 ^b^	N/A	-
	0.5	0 ^b^	0 ^b^	0 ^b^	0 ^b^	0 ^b^		
	1	0 ^b^	0 ^b^	0 ^b^	0 ^b^	0 ^b^		
Distilled water	0.1	0 ^b^	0 ^b^	0 ^b^	0 ^b^	0 ^b^	N/A	-
	0.5	0 ^b^	0 ^b^	0 ^b^	0 ^b^	0 ^b^		
	1	0 ^b^	0 ^b^	0 ^b^	0 ^b^	0 ^b^		
2% *α*-cypermethrin	0.1	0 ^b^	0 ^b^	0 ^b^	0 ^b^	0 ^b^	0.75	-
	0.5	0 ^b^	0 ^b^	0 ^b^	0 ^b^	0 ^b^		
	1	100 ^a^	100 ^a^	100 ^a^	100 ^a^	100 ^a^		
	ANOVA*_F_*_0.01_, D*f_total_*	**, 209	**, 209	**, 209	**, 209	**, 209		
	*p*-value	*p* < 0.01	*p* < 0.01	*p* < 0.01	*p* < 0.01	*p* < 0.01		

Mortality rates within a column with the same letters (a, b) do not differ significantly between the treatments (Tukey’s post hoc test, *p* < 0.01). ** for *p* < 0.01; N/A = not available.

## Data Availability

The original contributions presented in this study are included in the article. Further inquiries can be directed to the corresponding author.

## Abbreviations

The following abbreviations are used in this manuscript:

MI	Mortality Index
SV	Synergistic Value
IMV	Increased Mortality Value
BI	Biosafety Index

## References

[1] Geden C.J., Nayduch D., Scott J.G., Burgess E.R., Gerry A.C., Kaufman P.E., Thomson J., Pickens V., Machtinger E.T. (2021). House Fly (Diptera: Muscidae): Biology, Pest Status, Current Management Prospects, and Research Needs. J. Integr. Pest. Manag..

[2] Lu S., Miller N., Wilson A., Geden C.J., Stoffolano J.G., Ribeiro J.M.C. (2025). A Deep Insight into the Sialome of the House Fly, *Musca domestica*, Infected with the Salivary Gland Hypertrophy Virus (MdSGHV). Sci. Rep..

[3] Benelli G., Pavela R., Maggi F., Nkuimi Wandjou J.G., Yvette Fofie N.G.B., Koné-Bamba D., Sagratini G., Vittori S., Caprioli G. (2019). Insecticidal Activity of the Essential Oil and Polar Extracts from *Ocimum gratissimum* Grown in Ivory Coast: Efficacy on Insect Pests and Vectors and Impact on Non-Target Species. Ind. Crops Prod..

[4] Soonwera M., Moungthipmalai T., Puwanard C., Sittichok S., Sinthusiri J., Passara H. (2024). Adulticidal Synergy of Two Plant Essential Oils and Their Major Constituents against the Housefly *Musca domestica* and Bioassay on Non-Target Species. Heliyon.

[5] Sabahi Q., Hamiduzzaman M.M., Barajas-Pérez J.S., Tapia-Gonzalez J.M., Guzman-Novoa E. (2018). Toxicity of Anethole and the Essential Oils of Lemongrass and Sweet Marigold to the Parasitic Mite *Varroa destructor* and Their Selectivity for Honey Bee (*Apis mellifera*) Workers and Larvae. Psyche J. Entomol..

[6] Soonwera M., Sinthusiri J., Passara H., Moungthipmalai T., Puwanard C., Sittichok S., Murata K., Soonwera M., Sinthusiri J., Passara H. (2024). Combinations of Lemongrass and Star Anise Essential Oils and Their Main Constituent: Synergistic Housefly Repellency and Safety against Non-Target Organisms. Insects.

[7] Freeman J.C., Ross D.H., Scott J.G. (2019). Insecticide Resistance Monitoring of House Fly Populations from the United States. Pestic. Biochem. Physiol..

[8] Hafez A.M., Abbas N. (2023). Biological Fitness Cost, Demographic Growth Characteristics, and Resistance Mechanism in Alpha-Cypermethrin-Resistant *Musca domestica* (Diptera: Muscidae). Biology.

[9] Khan H.A.A., Shad S.A., Akram W. (2013). Resistance to New Chemical Insecticides in the House Fly, *Musca domestica* L., from Dairies in Punjab, Pakistan. Parasitol. Res..

[10] Wang J.-N., Hou J., Wu Y.-Y., Guo S., Liu Q.-M., Li T.-Q., Gong Z.-Y. (2019). Resistance of House Fly, *Musca domestica* L. (Diptera: Muscidae), to Five Insecticides in Zhejiang Province, China: The Situation in 2017. Can. J. Infect. Dis. Med. Microbiol..

[11] Lazarević-Pašti T., Milanković V., Tasić T., Petrović S., Leskovac A. (2025). With or Without You?—A Critical Review on Pesticides in Food. Foods.

[12] Ray S., Shaju S.T. (2023). Bioaccumulation of Pesticides in Fish Resulting Toxicities in Humans through Food Chain and Forensic Aspects. Environ. Anal. Health Toxicol..

[13] Siddiqui J.A., Fan R., Naz H., Bamisile B.S., Hafeez M., Ghani M.I., Wei Y., Xu Y., Chen X. (2023). Insights into Insecticide-Resistance Mechanisms in Invasive Species: Challenges and Control Strategies. Front. Physiol..

[14] Basavegowda N., Baek K.-H. (2022). Combination Strategies of Different Antimicrobials: An Efficient and Alternative Tool for Pathogen Inactivation. Biomedicines.

[15] Bouqellah N.A., Abdulmajeed A.M., Rashed Alharbi F.K., Mattar E., Al-Sarraj F., Abdulfattah A.M., Hassan M.M., Baazeem A., Al-Harthi H.F., Musa A. (2025). Optimizing Encapsulation of Garlic and Cinnamon Essential Oils in Silver Nanoparticles for Enhanced Antifungal Activity against *Botrytis cinerea* Pathogenic Disease. Physiol. Mol. Plant Pathol..

[16] Angane M., Swift S., Huang K., Butts C.A., Quek S.Y. (2022). Essential Oils and Their Major Components: An Updated Review on Antimicrobial Activities, Mechanism of Action and Their Potential Application in the Food Industry. Foods.

[17] Koul O. (2008). Essential Oils as Green Pesticides: Potential and Constraints. Biopestic. Int..

[18] George D.R., Finn R.D., Graham K.M., Sparagano O.A. (2014). Present and Future Potential of Plant-Derived Products to Control Arthropods of Veterinary and Medical Significance. Parasites Vectors.

[19] Demirak M.Ş.Ş., Canpolat E. (2022). Plant-Based Bioinsecticides for Mosquito Control: Impact on Insecticide Resistance and Disease Transmission. Insects.

[20] Koul O. (2008). Phytochemicals and Insect Control: An Antifeedant Approach. Crit. Rev. Plant Sci..

[21] Passara H., Sittichok S., Puwanard C., Sinthusiri J., Moungthipmalai T., Murata K., Soonwera M. (2024). Anise and Fennel Essential Oils and Their Combination as Natural and Safe Housefly Repellents. Insects.

[22] Passara H., Sittichok S., Sinthusiri J., Moungthipmalai T., Puwanard C., Murata K., Soonwera M., Passara H., Sittichok S., Sinthusiri J. (2024). Ovicidal Toxicity and Morphological Changes in Housefly Eggs Induced by the Essential Oils of Star Anise and Lemongrass and Their Main Constituents. Insects.

[23] Pavela R., Morshedloo M.R., Mumivand H., Khorsand G.J., Karami A., Maggi F., Desneux N., Benelli G. (2020). Phenolic Monoterpene-Rich Essential Oils from Apiaceae and Lamiaceae Species: Insecticidal Activity and Safety Evaluation on Non-Target Earthworms. Entomol. Gen..

[24] Sawatthum A. (2020). Role of Stingless Bee, *Tetragonula pegdeni* and European Honey Bee, *Apis mellifera* in the Pollination of Confectionery Sunflower. Thai J. Sci. Technol..

[25] Papa G., Maier R., Durazzo A., Lucarini M., Karabagias I.K., Plutino M., Bianchetto E., Aromolo R., Pignatti G., Ambrogio A. (2022). The Honey Bee *Apis mellifera*: An Insect at the Interface between Human and Ecosystem Health. Biology.

[26] Passara H., Moungthipmalai T., Laosinwattana C., Sittichok S., Murata K., Soonwera M. (2026). Terpenoid Mixtures as Repellents Against the American Cockroach: Their Synergy and Low Toxicity Against Non-Target Species. Insects.

[27] Babushok V., Linstrom P., Zenkevich I. (2011). Retention Indices for Frequently Reported Compounds of Plant Essential Oils. J. Phys. Chem. Ref. Data.

[28] Adams R. (2007). Identification of Essential Oil Components by Gas Chromatography/Mass Spectrometry.

[29] National Library of Medicine Pubchem: Explore Chemistry. https://pubchem.ncbi.nlm.nih.gov/.

[30] Soonwera M., Sittichok S. (2020). Adulticidal Activities of *Cymbopogon citratus* (Stapf.) and *Eucalyptus globulus* (Labill.) Essential Oils and of Their Synergistic Combinations against *Aedes aegypti* (L.), *Aedes albopictus* (Skuse), and *Musca domestica* (L.). Environ. Sci. Pollut. Res..

[31] Test Procedures for Insecticide Resistance Monitoring in Malaria Vector Mosquitoes. https://iris.who.int/items/45c8a14e-c5d2-4d98-9a35-525e41e9966a.

[32] OECD (1984). OECD Test No. 207: Earthworm, Acute Toxicity Tests. OECD Guidelines for the Testing of Chemicals.

[33] Wheeler M.W., Park R.M., Bailer A.J. (2006). Comparing Median Lethal Concentration Values Using Confidence Interval Overlap or Ratio Tests. Environ. Toxicol. Chem..

[34] Sittichok S., Passara H., Sinthusiri J., Soonwera M., Thongsaiklaing T., Morris J., Moungthipmalai T., Puwanard C., Jintanasirinurak S. (2025). Plant Essential Oils, *Trans*-Anethole and Eugenol, for Housefly Knockdown and Mortality. Int. J. Agric. Technol..

[35] Pavela R., Benelli G. (2016). Essential Oils as Ecofriendly Biopesticides? Challenges and Constraints. Trends Plant Sci..

[36] Mossa A.-T.H. (2016). Green Pesticides: Essential Oils as Biopesticides in Insect-pest Management. J. Environ. Sci. Technol..

[37] Gostin I.N., Popescu I.E., Gostin I.N., Popescu I.E. (2023). Evaluation of the Essential Oils Used in the Production of Biopesticides: Assessing Their Toxicity toward Both Arthropod Target Species and Beneficial Pollinators. Agriculture.

[38] Moungthipmalai T., Soonwera M. (2022). Adulticidal Activity against Housefly (*Musca domestica* L.; Muscidae: Diptera) of Eucalyptol, Limonene, and Their Combined Formulation. Int. J. Agric. Technol..

[39] Pavela R. (2008). Acute and Synergistic Effects of Some Monoterpenoid Essential Oil Compounds on the House Fly (*Musca domestica* L.). J. Essent. Oil Bear. Plants.

[40] Baker O.S., Norris E.J., Burgess E.R. (2023). Insecticidal and Synergistic Potential of Three Monoterpenoids against the Yellow Fever Mosquito, *Aedes aegypti* (Diptera: Culicidae), and the House Fly, *Musca domestica* (Diptera: Muscidae). Molecules.

[41] Benelli G., Pavela R., Iannarelli R., Petrelli R., Cappellacci L., Cianfaglione K., Heshmati Afshar F., Nicoletti M., Canale A., Maggi F. (2016). Synergized Mixtures of Apiaceae Essential Oils and Related Plant-Borne Compounds: Larvicidal Effectiveness on the Filariasis Vector *Culex quinquefasciatus* Say. Ind. Crops Prod..

[42] Chaubey M.K. (2021). Insecticidal Activities of *Anethum graveolens* L. and *Illicium verum* Hook. f. Essential Oils against *Sitophilus zeamais* Motschulsky. Rev. Cienc. Agrícolas.

[43] Sittichok S., Passara H., Sinthusiri J., Moungthipmalai T., Puwanard C., Murata K., Soonwera M. (2024). Synergistic Larvicidal and Pupicidal Toxicity and the Morphological Impact of the Dengue Vector (*Aedes aegypti*) Induced by Geranial and Trans-Cinnamaldehyde. Insects.

[44] Serrão J.E., Plata-Rueda A., Martínez L.C., Zanuncio J.C. (2022). Side-Effects of Pesticides on Non-Target Insects in Agriculture: A Mini-Review. Sci. Nat..

[45] Abbas N., Hafez A.M. (2023). Alpha-Cypermethrin Resistance in *Musca domestica*: Resistance Instability, Realized Heritability, Risk Assessment, and Insecticide Cross-Resistance. Insects.

[46] Tak J.-H., Jovel E., Isman M.B. (2017). Synergistic Interactions among the Major Constituents of Lemongrass Essential Oil against Larvae and an Ovarian Cell Line of the Cabbage Looper, *Trichoplusia ni*. J. Pest. Sci..

[47] Shahriari M., Zibaee A., Sahebzadeh N., Shamakhi L. (2018). Effects of *α*-Pinene, *Trans*-Anethole, and Thymol as the Essential Oil Constituents on Antioxidant System and Acetylcholine Esterase of *Ephestia kuehniella* Zeller (Lepidoptera: Pyralidae). Pestic. Biochem. Physiol..

[48] Silvério M.R.S., Espindola L.S., Lopes N.P., Vieira P.C., Silvério M.R.S., Espindola L.S., Lopes N.P., Vieira P.C. (2020). Plant Natural Products for the Control of *Aedes aegypti*: The Main Vector of Important Arboviruses. Molecules.

[49] Aungtikun J., Soonwera M., Sittichok S. (2021). Insecticidal Synergy of Essential Oils from *Cymbopogon citratus* (Stapf.), *Myristica fragrans* (Houtt.), and *Illicium verum* Hook. f. and Their Major Active Constituents. Ind. Crops Prod..

[50] Zhang Y., Wang Y., Zhao N., Lun X., Zhao C., Liu Q., Meng F. (2024). Long-Term Trends in Housefly (*Musca domestica* L.) Insecticide Resistance in China. Pestic. Biochem. Physiol..

[51] Hilliou F., Chertemps T., Maïbèche M., Goff G.L. (2021). Resistance in the Genus Spodoptera: Key Insect Detoxification Genes. Insects.

[52] Park Y.-L., Tak J.-H. (2016). Essential Oils for Arthropod Pest Management in Agricultural Production Systems. Essential Oils in Food Preservation, Flavor and Safety.

[53] Papachristos D.P., Stamopoulos D.C. (2003). Selection of *Acanthoscelides obtectus* (Say) for Resistance to Lavender Essential Oil Vapour. J. Stored Prod. Res..

[54] Farag M.R., Alagawany M., Bilal R.M., Gewida A.G.A., Dhama K., Abdel-Latif H.M.R., Amer M.S., Rivero-Perez N., Zaragoza-Bastida A., Binnaser Y.S. (2021). An Overview on the Potential Hazards of Pyrethroid Insecticides in Fish, with Special Emphasis on Cypermethrin Toxicity. Animals.

[55] Kašuba V., Lovaković B.T., Vrdoljak A.L., Katić A., Kopjar N., Micek V., Milić M., Pizent A., Želježić D., Žunec S. (2022). Evaluation of Toxic Effects Induced by Sub-Acute Exposure to Low Doses of *α*-Cypermethrin in Adult Male Rats. Toxics.

[56] Kaur R., Singh J. (2021). Toxicity, Monitoring, and Biodegradation of Cypermethrin Insecticide: A Review. Nat. Environ. Pollut. Technol..

[57] Pashte V.V., Patil C.S. (2018). Toxicity and Poisoning Symptoms of Selected Insecticides to Honey Bees (*Apis mellifera mellifera* L.). Arch. Biol. Sci..

[58] Chibee G.U., Ojelabi O.M., Fajana H.O., Akinpelu B.A., Kehinde T.O., Awodiran O.M., Obuotor E.M., Owojori O.J. (2021). Effects of Cypermethrin as a Model Chemical on Life Cycle and Biochemical Responses of the Tropical Stingless Bee *Meliponula bocandei* Spinola, 1853. Environ. Adv..

[59] Fent K., Schmid M., Christen V. (2020). Global Transcriptome Analysis Reveals Relevant Effects at Environmental Concentrations of Cypermethrin in Honey Bees (*Apis mellifera*). Environ. Pollut..

[60] Yang Y., Ma S., Liu F., Wang Q., Wang X., Hou C., Wu Y., Gao J., Zhang L., Liu Y. (2020). Acute and Chronic Toxicity of Acetamiprid, Carbaryl, Cypermethrin and Deltamethrin to *Apis mellifera* Larvae Reared In Vitro. Pest. Manag. Sci..

[61] Shilpakar O., Karki B. (2021). Cypermethrin Poisoning Manifesting with Prolonged Bradycardia: A Case Report. Toxicol. Rep..

[62] Shuklan P., Raj A., Chauhan K., Madan P., Rani S. (2023). Systematic Toxicity of Cypermethrin and Alterations in Behavior of Albino Rats. ACS Omega.

[63] Tremongkoltip A., Pengpumkiat S., Kongtip P., Nankongnab N., Siri S., Woskie S. (2023). Urinary Cypermethrin Metabolites among Conventional and Organic Farmers in Thailand. Toxics.

[64] Caren J., Zhu Y.-C., Read Q.D., Du Y. (2025). Risk Assessment of Effects of Essential Oils on Honey Bees (*Apis mellifera* L.). Insects.

[65] Conti B., Flamini G., Cioni P.L., Ceccarini L., Macchia M., Benelli G. (2014). Mosquitocidal Essential Oils: Are They Safe against Non-Target Aquatic Organisms?. Parasitol. Res..

[66] Benelli G., Pavela R., Petrelli R., Cappellacci L., Canale A., Senthil-Nathan S., Maggi F. (2018). Not Just Popular Spices! Essential Oils from *Cuminum cyminum* and *Pimpinella anisum* Are Toxic to Insect Pests and Vectors without Affecting Non-Target Invertebrates. Ind. Crops Prod..

[67] Pavela R. (2018). Essential Oils from *Foeniculum vulgare* Miller as a Safe Environmental Insecticide against the *Aphid myzus* Persicae Sulzer. Environ. Sci. Pollut. Res..

[68] Asif M., Yehya A.H.S., Al-Mansoub M.A., Revadigar V., Ezzat M.O., Khadeer Ahamed M.B., Oon C.E., Murugaiyah V., Abdul Majid A.S., Abdul Majid A.M.S. (2016). Anticancer Attributes of *Illicium verum* Essential Oils against Colon Cancer. S. Afr. J. Bot..

[69] Zhu L., Luo Y., Xiao J., Hao E., Wei J., Zhao J., Yao C., Wang Y., Luo H. (2024). Star Anise (*Illicium verum* Hook. f.): Dual Therapeutic and Nutritional Potential in Food and Medicine. Acupunct. Herb. Med..

[70] Solon I.G., Santos W.S., Branco L.G.S. (2025). Citral as an Anti-Inflammatory Agent: Mechanisms, Therapeutic Potential, and Perspectives. Pharmacol. Res.-Nat. Prod..

[71] Sharma S., Habib S., Sahu D., Gupta J. (2020). Chemical Properties and Therapeutic Potential of Citral, a Monoterpene Isolated from Lemongrass. Med. Chem..

